# Psychological Typhoon Eye in the 2008 Wenchuan Earthquake

**DOI:** 10.1371/journal.pone.0004964

**Published:** 2009-03-23

**Authors:** Shu Li, Li-Lin Rao, Xiao-Peng Ren, Xin-Wen Bai, Rui Zheng, Jin-Zhen Li, Zuo-Jun Wang, Huan Liu

**Affiliations:** Institute of Psychology, Chinese Academy of Sciences, Beijing, China; Aga Khan University Hospital, Pakistan

## Abstract

**Background:**

On May 12, 2008, an earthquake measuring 8.0 on the Richter scale jolted Wenchuan, China, leading to 69,227 deaths and 374,643 injured, with 17,923 listed as missing as of Sept. 25, 2008, and shook the whole nation. We assessed the devastating effects on people's post-earthquake concern about safety and health.

**Methodology/Principal Findings:**

From June 4 to July 15, 2008, we surveyed a convenience sample of 2,262 adults on their post-earthquake concern about safety and health. Residents in non-devastated areas (Fujian and Hunan Provinces, and Beijing) and devastated areas (Sichuan and Gansu Provinces) responded to a questionnaire of 5 questions regarding safety measures, epidemic disease, medical workers, psychological workers, and medication. The ANOVAs showed a significant effect of residential devastation level on the estimated number of safety measures needed, the estimated probability of the outbreak of an epidemic, and the estimated number of medical and psychological workers needed (Ps<0.001). The post-earthquake concern decreased significantly as the level of residential devastation increased. Because of the similarity with the meteorological phenomenon of the eye of a typhoon, we dubbed these findings a “Psychological Typhoon Eye”: the closer to the center of the devastated areas, the less the concern about safety and health a resident felt.

**Conclusions/Significance:**

Contrary to common perception and ripple effect that the impact of an unfortunate event decays gradually as ripples spread outward from a center, a “Psychological Typhoon Eye” effect was observed where the post-earthquake concern was at its lowest level in the extremely devastated areas. The resultant findings may have implications for Chinese governmental strategies for putting “psychological comfort” into effect.

## Introduction

A catastrophic earthquake measuring 8.0 on the Richter scale jolted China's Sichuan Province on May, 12, 2008. Official figures (as of Sept. 25, 2008, 12:00 CST) stated that 69,227 were confirmed dead and 374,643 injured with 17,923 listed as missing [1]. It was the first time in history that China declared three days (May 19 to May 21) of national mourning for the earthquake victims. The Olympic torch relay was also suspended to express mourning. The severe disaster attracted the attention of the whole Chinese nation. The concern was so deep that blood-donation is now overwhelmingly seen as a “helpful enough” way to express one's love [2]. The enthusiasm of the masses, however, turned out to be the first example of “overestimation of aid needed” – blood banks in other parts of the country received so many donations in the first few days after the earthquake that they had to turn people away [3], [4].

We conducted a study to assess whether people's post-earthquake concern about safety and health varied gradually with devastation level from the center of the disaster area. We focused on the potential differences of post-earthquake concern between residents in the non-devastated areas and those in the devastated areas.

## Methods

### Data Collection and Sample

The survey period was three to eight weeks after the earthquake – from June 4 to July 15, 2008. The study was part of the Emergency Project to Provide Psychological Assistance in Wenchuan Earthquake Areas (No. KKCX1-YW-05) approved by the Institutional Review Board of the Institute of Psychology, Chinese Academy of Sciences. Because the protocol was judged to pose low risk, oral consent was recommended and obtained from study participants.

**Figure 1:**
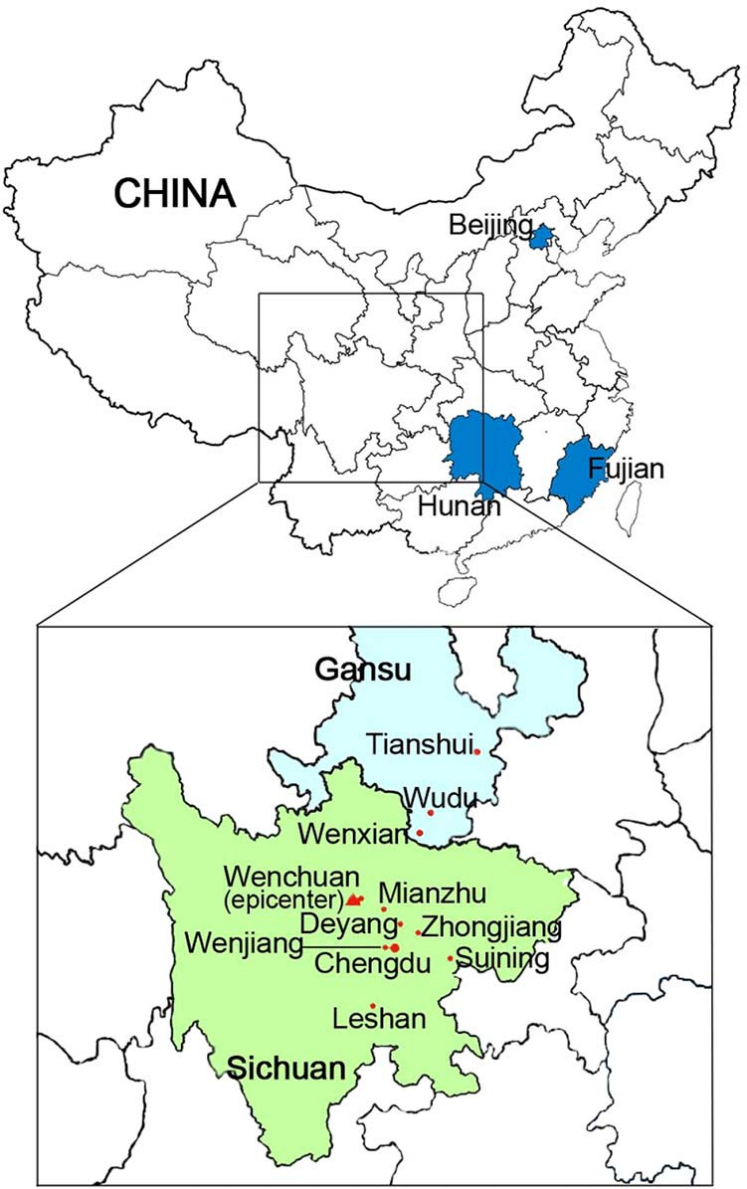
Sampling Frame in Relation to the Epicenter. The sampling frame includes three non-devastated areas indicated by blue shading (Fujian Province, Hunan Province and Beijing) and eleven devastated areas indicated by red dots (Wenchuan, Chengdu, Wenjiang, Suining, Leshan, Deyang, Zhongjiang, and Mianzhu in Sichuan Province, and Tianshui, Wenxian and Wudu in Gansu Province). This figure was plotted by Li-Rong Zhang.

A convenience sample of residents in non-devastated areas as well as devastated areas was surveyed. Five hundred forty two adults living in Fujian Province (South China), Hunan Province (Central South China) and Beijing (North China) were selected to represent a sample of residents in non-devastated areas. For devastated areas, the sampling frame consisted of 1,720 adults living in eight administrational areas in Sichuan Province and three administrational areas in Gansu Province (Figure 1).

Entry criteria included an age of at least 18 years, literacy, and a willingness to provide personal contact information. Respondents were paid a small fee (or a small present, such as soap, a towel, and washing powder) for each completed questionnaire. Demographic data on the 2,262 respondents are presented in Table 1.

**Table 1:** Demographic Characteristics of Residents in Non-devastated Areas and Devastated Areas.

Characteristic	Residence in Non-devastated Areas	Residence in Devastated Areas	Total
	(N = 542)	(N = 1720)	(N = 2262)
Age (yr)			
Mean	35.86±12.58	31.31±8.52	32.40±9.83
Median	33	30	30
	No. of Respondents (%)		
Sex			
Male	275 (50.7)	666 (38.7)	941 (41.6)
Female	261 (48.2)	1049 (61.0)	1310 (57.9)
Unknown	6 (1.1)	5 (0.3)	11 (0.5)
Education			
Below high-school graduate	57 (10.5)	427 (24.8)	484 (21.4)
High-school graduate	122 (22.5)	618 (35.9)	740 (32.7)
Beyond high-school graduate	354 (65.3)	668 (38.8)	1022 (45.2)
Unknown	9 (1.7)	7 (0.4)	16 (0.7)
Occupation			
Civil servant	51 (9.4)	37 (2.2)	88 (3.9)
Employee of public institutions	53 (9.8)	207 (12.0)	260 (11.5)
Enterprises employee	207 (38.2)	724 (42.1)	931 (41.2)
Medical worker	44 (8.1)	75 (4.4)	119 (5.3)
Teacher	73 (13.5)	193 (11.2)	266 (11.8)
Farmer	52 (9.6)	118 (6.9)	170 (7.5)
Student	11 (2.0)	45 (2.6)	56 (2.5)
Other	40 (7.4)	306 (17.8)	346 (15.3)
Unknown	11(2.0)	15 (0.9)	26 (1.1)

### Key Measures

“I am always scared. Can you prescribe some medication to help me get rid of fear?”

—— LI Miao-Yu, an 8-year-old girl from Ziping Village, Dujiangyan.

The above quotation comes from an on-the-spot report published on June 1, 2008, in *Southern People Weekly* [5]. The report narrates the true story of a little girl from an extremely devastated area (Dujiangyan) who naively longs for a medication that can heal the psychological trauma of the mass disaster. In the same vein, we developed a question to assess people's post-earthquake concern about psychological health by taking hypothetical medication as an index, which was once used to evaluate anticipated and experienced emotion by Wilson and coworkers [6]. The respondents in quake areas were asked to state a dosage of medication for an earthquake victim. The higher the dosage prescribed, the more severe the trauma presumably perceived.

The other four questions were also developed to assess the concern about safety and health. These questions were distributed to residents in both devastated and non-devastated areas. They were mainly represented in a frequency-estimate format, which was proved to reduce judgmental errors and biases [7], [8]. The respondents were asked to indicate the number of relief workers (medical and psychological) needed, the probability of an outbreak of an epidemic, and the number of safety measures needed. The higher mean estimated the higher concern about safety and health presumably perceived. The details of these five questions are shown in Table 2. The questions were interspersed among other questions about the measurement of “Psychological Harmony” in a lengthy questionnaire.

**Table 2:** The Post-earthquake Concern about Safety and Health Assessed on the Response to the Five Questions.*

Question	Non-devastated Area		Devastated area						P Value
			Slightly		Moderately		Extremely		
	No.	Response	No.	Response	No.	Response	No.	Response	
1. What is the probability (0–100%) that an epidemic disease will be widespread in the post-earthquake areas? †	517	36.77±27.21	903	32.49±25.04	354	29.36±23.62	429	27.52±24.06	<0.001
2. How many times (out of 100 aftershocks) would residents in the earthquake areas need to take safety measures? †	488	50.49±40.26	879	31.01±36.19	351	24.48±32.68	418	25.48±33.35	<0.001
3. How many Medical Doctors are needed for every 1000 residents in the earthquake areas? †	509	117.99±188.21	892	130.46±210.55	351	98.15±174.08	422	75.95±139.40	<0.001
4. How many Psychological Workers are needed for every 1000 residents in the earthquake areas? †	510	131.43±226.88	900	149.56±252.12	354	113.71±226.85	422	77.25±177.18	<0.001
5. Suppose that there is a medication which can heal the psychological wounds of mass disaster without side effects such as nausea or anaphylaxis. What dose of the medication should be prescribed for an earthquake victim (up to 100 mg daily)? † ‡			882	65.46±32.62	344	62.51±31.14	404	59.00±32.60	0.004

* Plus–minus values are means±SD. The characteristics were compared by one way analysis of variance.

† Numbers may not add up to 2262 because not all the respondents answered all the questions.

‡ The estimated dosage more than 100 was coded as the maximum value (100 mg daily).

To determine the level of residential devastation from all devastated areas, we asked respondents to classify their residency in one of three categories: slightly devastated, moderately devastated, and extremely devastated. We assessed the relationship between post-earthquake concern and the level of residential devastation.

### Statistical Analysis

The data were analyzed on a personal computer with an SPSS statistical software package (version 16.0 for Windows). Comparison of the different residential devastation levels was completed with the use of one-way analysis of variance. Two-way analysis of variance (involving two factors: type of relief worker, and residential devastation level) followed by Tamhane's post hoc tests was used to compare the mean estimated number of relief workers needed. No imputation of missing values was performed. All P values are two-sided; a P value of less than 0.05 was considered statistically significant.

## Results

### Sample

Of the 1,720 residents surveyed in the devastated areas, 53.4 percent (918) reported their residency as slightly devastated, 21 percent (362) moderately devastated, and 25.6 percent (440) extremely devastated.

### Post-earthquake concern about safety and health

Table 2 presents the result of residents' reactions to the five questions posed.

The estimated number of medical doctors needed for every 1,000 residents in the earthquake areas was 117.99±188.21 for residents in the non-devastated areas, 130.46±210.55 for residents in the slightly devastated areas, 98.15±174.08 for residents in the moderately devastated areas, and 75.95±139.40 for residents in the extremely devastated areas. The estimated numbers of psychological workers needed for every 1,000 residents in the earthquake areas were 131.43±226.88 for residents in the non-devastated areas, 149.56±252.12 for residents in the slightly devastated areas, 113.71±226.85 for residents in the moderately devastated areas, and 77.25±177.18 for residents in the extremely devastated areas. There was a significant difference in the type of relief worker (P = 0.008, for a two-way analysis of variance), indicating that the estimated need for psychological workers was greater than that for medical doctors; and a significant effect of residential devastation level (P<0.001), indicating that the estimated numbers of relief workers decreased as the residential devastation level increased. There was no significant interaction between the type of relief worker and the residential devastation level (P = 0.287). Residents in the extremely devastated areas reported a lower number of relief workers needed for every 1,000 residents in earthquake areas than residents in the slightly devastated and non-devastated areas (Ps<0.001), with no significant difference between the latter two groups (P = 0.645).

The estimated probability of an outbreak of an epidemic showed a significant decrease associated with increased residential devastation level (P<0.001 by one way analysis of variance). Residents in the non-devastated areas indicated a higher probability of an outbreak of an epidemic (for a mean of 36.77) than residents in each of the three devastated areas (for a mean of 32.49, 29.36, 27.52, respectively; Ps≤0.002, for Tamhane's test). Residents in the slightly-devastated areas indicated a higher probability of an outbreak of an epidemic than residents in the extremely devastated areas (P = 0.003, for Tamhane's test) (Table 2).

There was a similar trend toward a smaller estimated number of safety measures needed with an increasing level of residential devastation (P<0.001 by one way analysis of variance). Residents in the non-devastated areas indicated a greater number of safety measures needed (for a mean of 50.49) than those in each of the three devastated areas (for a mean of 31.01, 24.48, 25.48, respectively; Ps<0.001, for Tamhane's tests). Residents in the slightly-devastated areas indicated a greater number of safety measures behavior needed than their counterparts in the moderately (P = 0.013, for Tamhane's test) and extremely devastated areas (P = 0.04, for Tamhane's test) (Table 2).

There were significant differences in daily dose of medication demanded for an earthquake victim among the three groups of residents in devastated areas (P = 0.004 by one way analysis of variance) (Table 2). Residents in the extremely devastated areas indicated the lowest daily dose of medication demanded for an earthquake victim (for a mean of 59.00), while residents in the slightly devastated areas indicated the highest daily dose of medication (for a mean of 65.46) (Table 2).

## Discussion

In contrast to the common perception and ripple effect [9] that the impact of an unfortunate event attenuates over distance, just as ripples spread outward, our findings in this study revealed that residents felt less concern when their residential devastation level increased from non-devastated to slightly devastated, to moderately devastated, and further towards extremely devastated.

Chen *et al.* found that in the 1999 Taiwan Chi-Chi Earthquake the number of physicians in the emergency department was insufficient in the initial hours after the earthquake [10]. We found that in the 2008 Wenchuan Earthquake the estimated number of psychological workers demanded was greater than that of medical doctors across all the regions surveyed. The uneven demand implied that the number of psychological workers might be insufficient (the ratio of current members of Chinese Medical Association to that of Chinese Psychological Society is about 430,000∶6,000 [11], [12]). The higher demand for psychological workers was possibly due to people's increasing concern about psychological health but with limited knowledge of the work of “psychological workers” and that of other volunteers. The increasing concern about psychological health was very likely driven by the Chinese government's recent appeal to provide more material support and psychological comfort to quake survivors [13] or by Hu Jintao's report delivered at the 17th National Congress of the Communist Party of China (CPC), in which an academic term “*psychological counseling*” appeared for the first time in official CPC documents [14].

In a study of stress reactions after the disaster of September 11, 2001, Schuster *et al.* suggested that even people far from the attack would have trauma-related symptoms of stress [15]. Our findings suggested that people far from the earthquake area (more remote) were more likely to have a high estimation of their post-earthquake concern. Such a high estimation was expressed in terms of the estimated number of relief workers needed, the estimated probability of outbreak of an epidemic, and the estimated number of safety measures needed.

We also found that, for the sample of residents in devastated areas (Sichuan and Gansu Provinces), those who considered their residency extremely, but not slightly or moderately, devastated indicated a minimum dose for a hypothetical medication. It seems that the strongest resilience is achieved by those who reside in an unexpected place.

Taken together, these results suggest a “Psychological Typhoon Eye” effect [16], where residents in extremely devastated areas were found to have the lowest post-earthquake concern about safety and health. This is thus analogous to the meteorological phenomenon of the eye of a typhoon, referring to a region of mostly calm weather found at the center of a strong tropical cyclone.

Similar interesting findings have also appeared in risk perception literature. In studying the effect of distance upon risk perception, Guedeney and Mendel reported that in a local attitude survey of a nuclear power station in France anxiety is lower among those living near the nuclear reactor [17]. Maderthaner *et al.* also found that people living far from the nuclear research reactor perceived it to be riskier than the nearer residents [18].

There are several possible/alternative explanations for this effect. One is the “psychological immunization” theory, which assumes that resistance to adverse life events is naturally acquired through repeated exposure [19]. Residents in devastated quake areas were supposed to be given an increased psychological immunity to the severe disaster by natural exposure to hazard stimuli.

Another possible, but more fundamental explanation, of this effect might be Festinger's Theory of Cognitive Dissonance, which states that cognitive dissonance is an uncomfortable psychological state in which the individual experiences two incompatible beliefs or cognitions [20]. An individual is motivated by the attendant discomfort to act in such a manner so as to reduce dissonance. In the present case, people living near the eye of the devastated areas are presumably more likely than people living far away to believe that the risk is low at the site. This is because the nearby residents tend to feel cognitive dissonance – there is a post-earthquake risk and they are continuing to live nearby and, therefore, want to restore consistency or consonance. The main way of reducing dissonance, if they cannot change their residence, is to change their beliefs and attitudes about living in a potential risky situation.

A potential limitation of our study is that we used a convenience sample rather than a population-based sample. Ideally a population-based random sample should have been collected at least in the non-devastated areas (Fujian Province, Hunan Province, and Beijing). In the devastated areas, however, a convenience sample may have been the only feasible method of gathering potential respondents, that is to say, we were able to interview only those individuals who were settled in Red Cross tents.

If successful, this study should aid risk analysis and policy-making by providing a basis for understanding and predicting public response to disasters. The experience of the Wenchuan Earthquake may have implications for governmental strategies for responding to other major disasters, including heavy blizzards and hand, foot, and mouth disease (HFMD), from which China suffered during the first half of 2008. Future research in earthquake areas should determine the functional role of the factors (remote in time and kinship) that were associated with post-earthquake concern about safety and health in our study.
